# Clinical Utility of AI-Enabled CT-to-MRI Translation for Degenerative Spinal Disorders: A Retrospective Reader Study

**DOI:** 10.3390/diagnostics16111589

**Published:** 2026-05-22

**Authors:** Taehwan Kim, Hanul Gong, Hyung-Youl Park, Yeo Dong Yoon

**Affiliations:** 1 AI R&D Center, Polestar Healthcare Inc., 331 Gangnam-daero, Seocho-gu, Seoul 06627, Republic of Korea; taehwan@polestarhc.com (T.K.); ghanul@polestarhc.com (H.G.); 2 Department of Orthopedic Surgery, Eunpyeong St. Mary’s Hospital, College of Medicine, The Catholic University of Korea, Seoul 03312, Republic of Korea; matrixbest@naver.com

**Keywords:** CT-to-MRI translation, degenerative spinal disorders, retrospective reader study

## Abstract

**Background/Objectives**: MRI is preferred for disc-related assessment in suspected degenerative spinal disorders, but it may be delayed, unavailable, or contraindicated; in such cases, CT findings often guide initial decisions. To address these limitations in MRI accessibility, we developed Dr.Magic (DRM-S-01), an AI-enabled CT-to-MRI translation system that converts non-contrast spine CT into MRI-like translated images ($\hat{T}$-MRI), and conducted a retrospective reader study to assess its clinical utility. **Methods**: Ninety-two paired CT/MRI examinations were independently reviewed by three board-certified radiologists under three conditions: MRI-only, CT-only, and CT augmented with $\hat{T}$-MRI. MRI-only interpretation served as the per-reader reference standard. **Results**: CT augmented with $\hat{T}$-MRI improved diagnostic accuracy for disc-related assessment versus CT-only for all readers (34.78% to 72.83%, 42.39% to 77.17%, and 40.22% to 69.57%; all *p* < 0.01), increasing mean accuracy from 39.13% ± 3.20% to 73.19% ± 3.12%. Inter-reader agreement also improved (Fleiss’ κ: 0.5617 to 0.6621; observed agreement: 0.6630 to 0.8116). **Conclusions**: Overall, these findings suggest that augmenting CT interpretation with $\hat{T}$-MRI may improve diagnostic performance and reading consistency when timely MRI is not feasible. In our implementation, Dr.Magic completed $\hat{T}$-MRI translation in a median of 10.90 s per CT examination (IQR, 10.39–11.79), supporting practical use within CT-based workflows.

## 1. Introduction

Degenerative spinal disorders, including intervertebral disc degeneration and disc herniation, impose a significant and growing health burden worldwide. Recent epidemiological analyses estimate that approximately 5% of the global population—over 400 million people—suffer from symptomatic degenerative spine conditions, with lumbar disc degeneration being a major contributor [1]. Degenerative imaging findings are common across the adult lifespan, including among asymptomatic individuals, and become more prevalent with increasing age [2]. In South Korea, the trends are similar—a nationwide study reported that the annual incidence of herniated lumbar disc approaches 0.8–0.9% of the population, corresponding to nearly half a million new cases annually, with particularly high rates in older adults [3]. These data underscore the enormous clinical burden of degenerative spinal disorders in aging populations.

Medical imaging is central to the clinical diagnosis of degenerative spinal disorders. Because disc-related abnormalities often correlate imperfectly with symptoms and physical examination findings, clinicians rely on imaging to confirm pathology and localize the affected spinal level [4,5]. Computed tomography (CT) and magnetic resonance imaging (MRI) are the two most commonly used modalities in this setting, providing complementary information. CT is widely available, rapid to acquire, and offers excellent visualization of bony anatomy, supporting assessment of vertebral alignment and degenerative bony changes. However, its limited soft-tissue contrast restricts reliable evaluation of intervertebral discs and associated degenerative changes [6]. In contrast, MRI is widely regarded as the reference standard for evaluating disc pathology. It enables detailed assessment of disc morphology, signal intensity changes associated with degeneration, and disc herniation, thereby supporting accurate diagnosis, level localization, and clinical decision-making [4,7]. Despite these advantages, MRI has several practical limitations, including higher cost, longer acquisition times, constrained availability in some clinical settings, and contraindications such as claustrophobia or non-MRI-compatible implanted devices, including pacemakers [8,9,10]. When MRI is unavailable, delayed, or contraindicated, CT may be used as an alternative or interim approach despite MRI’s advantages for disc-related assessment [11]. In such situations, clinicians may need to rely on CT with limited confidence for disc-related findings. This practical gap—CT’s accessibility versus MRI’s superior soft-tissue assessment—highlights the need for approaches that can provide MRI-relevant disc information at the time of CT.

Recent advances in artificial intelligence (AI), particularly in deep learning-based image-to-image translation, have accelerated research on modality translation in medical imaging [12,13,14,15]. These methods aim to augment clinical interpretation by transforming information from routinely acquired imaging—most notably CT—into representations that better reflect soft-tissue characteristics typically assessed on MRI. In degenerative spinal disorders, these advances motivate the development of CT-to-MRI translation. CT is often obtained at the initial assessment, whereas MRI, despite its diagnostic superiority for disc-related pathology, may be delayed or unavailable. By translating CT into MRI-like images, AI-based approaches may provide additional soft-tissue information relevant to disc morphology and degenerative changes at the initial assessment. Importantly, the goal of CT-to-MRI translation is not to replace conventional MRI, but to augment CT-based interpretation by providing additional soft-tissue information that may support screening or triage, including prioritization for subsequent MRI when access is limited [16,17,18]. Overall, in degenerative spinal disorders, AI-enabled CT-to-MRI translation may help bridge the gap between the availability of CT at initial assessment and MRI’s advantages in soft-tissue evaluation when timely MRI is not feasible.

Accordingly, we developed Dr.Magic (DRM-S-01), an AI-enabled CT-to-MRI translation system for degenerative spinal disorders. Dr.Magic translates routinely acquired non-contrast spine CT images into MRI-like translated images ($\hat{T}$-MRI), including T1-weighted-like and T2-weighted-like representations. $\hat{T}$-MRI depicts disc morphology and degenerative changes while maintaining CT-consistent anatomy, providing soft-tissue information that is limited on CT to support disc-related interpretation. In this retrospective reader study, we evaluated whether CT interpretation supported by $\hat{T}$-MRI could improve diagnostic assessment of degenerative spinal disorders compared with CT-only interpretation, using MRI-only interpretation as the reference standard.

## 2. Materials and Methods

### 2.1. Study Design

This study was designed as a retrospective reader study to assess the clinical utility of an AI-enabled CT-to-MRI translation system (Dr.Magic (Polestar Healthcare Co., Ltd., Seoul, Republic of Korea, https://en.polestarhc.com/drmagic (accessed on 19 May 2026))) for degenerative spinal disorders. Imaging data were retrospectively collected at a single institution from clinically acquired CT and MRI examinations. This study was approved by the Institutional Review Board of Eunpyeong St. Mary’s Hospital, The Catholic University of Korea (IRB No. PC25DSSS0188), and the requirement for informed consent was waived. All procedures were conducted in accordance with the principles of the Declaration of Helsinki. Three board-certified radiologists participated in the reader study. The detailed reading procedure and evaluation protocol are described below.

### 2.2. Study Population

Patients were retrospectively identified from clinically acquired CT and MRI examinations performed between 1 March 2019, and 30 August 2025. Eligible cases included adult patients (≥19 years) who underwent both non-contrast CT and MRI for the evaluation of degenerative spinal disorders. To reduce the likelihood of substantial clinical change between examinations, only cases in which the interval between CT and MRI examinations was within 6 months were included.

The predefined exclusion criteria were as follows: prior use of the imaging data during AI model development; missing or non-interpretable CT or MRI examinations; spinal surgery or intervention between the CT and MRI examinations; substantial image distortion due to metallic artifacts; deviation from the specified non-contrast imaging protocol; and insufficient image quality precluding reliable interpretation.

**Figure 1:**
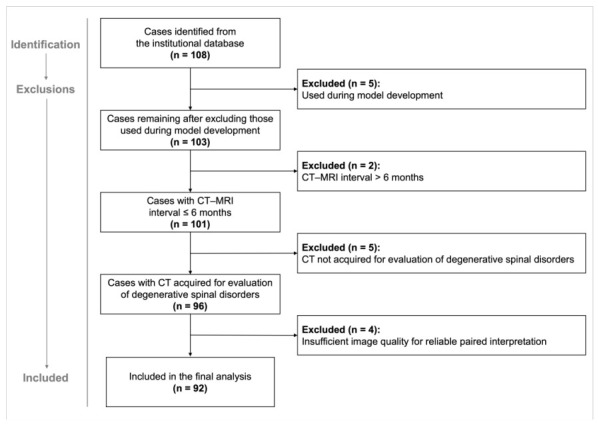
Flow diagram of case selection for the retrospective reader study. Patients who underwent both non-contrast CT and MRI for evaluation of degenerative spinal disorders were screened, and 92 patients were included in the final analysis after application of the predefined eligibility and exclusion criteria.

These criteria were intended to exclude cases with substantial clinical change, imaging conditions that could impair reliable comparison between CT and MRI, or inadequate image quality precluding confident interpretation. In our study, exclusions in the final cohort were applied according to the case-screening workflow summarized in Figure 1, and no cases were excluded for reasons other than those shown.

A total of 92 patients were included in the final analysis. The mean age of the study population was 68.9 ± 9.6 years, and 38 patients were male and 54 were female. The median interval between CT and MRI examinations was 17 days (interquartile range [IQR], 1–37.5 days; range, 0–175 days). Patient characteristics are summarized in Table 1.

### 2.3. Imaging Acquisition

CT and MRI examinations were obtained as part of routine clinical practice using non-contrast protocols. All CT studies were performed using scanners from Siemens Healthineers (Erlangen, Germany). Tube voltage was 150 kVp in 49/92 (53.3%), 120 kVp in 36/92 (39.1%), and 100 kVp in 7/92 (7.6%). Images were reconstructed using Br40s in 83/92 (90.2%) and Br44d in 9/92 (9.8%) with a slice thickness of 2 mm. Exposure time was 1000 ms in 83/92 (90.2%) and 625 ms in 9/92 (9.8%). Tube loading had a median of 266 mAs (IQR, 220–340) with a range of 173–681 mAs. The volume CT dose index had a median of 11.8 mGy (IQR, 9.7–14.8) with a range of 1.6–22.8 mGy. MRI examinations were performed predominantly on 3.0 T systems, with a single examination acquired on a 1.5 T system. MRI scanners were predominantly from Siemens Healthineers, with one examination performed on a GE Healthcare system. All MRI examinations were non-contrast studies and followed routine clinical spine protocols for degenerative spinal disorder evaluation. The protocol included sagittal and axial T1- and T2-weighted sequences acquired as 2D spin-echo-based imaging. Slice thickness was 4.0 mm in 89/92 examinations (range, 3.0–4.5 mm). In-plane pixel spacing had a median of 0.45 mm (IQR 0.281–0.45; range, 0.208–0.45 mm). For each patient, CT and MRI examinations were matched at the patient level. Both sagittal and axial images were available for evaluation in all cases. Scout or localizer images acquired for positioning were excluded from evaluation.

### 2.4. AI-Enabled CT-to-MRI Translation System

Dr.Magic (DRM-S-01) is an AI-enabled CT-to-MRI translation system that translates non-contrast spine CT into $\hat{T}$-MRI for use in the assessment of degenerative spinal disorders. The AI model was locked prior to this study, and the study data were not used for any model updates, including training, fine-tuning, or parameter calibration.

Dr.Magic takes non-contrast CT examinations as input and provides $\hat{T}$-MRI images that emphasize disc morphology and degenerative changes. The system can output T1-weighted-like and/or T2-weighted-like $\hat{T}$-MRI images. These translated images are provided in the same spatial geometry as the input CT, preserving coordinate alignment while conveying additional imaging information. The training dataset consisted of 250 patients with paired CT/MRI examinations and was split into training (*n* = 222) and internal validation (*n* = 28) sets. All training cases included non-contrast spine CT and corresponding non-contrast MRI that contained both T1- and T2-weighted sequences acquired in sagittal and axial planes. All training/validation cases were collected prior to this study and had no overlap with the 92 cases used for clinical evaluation. The underlying model was trained using a CycleGAN-based framework [19]. The generator architecture was based on a U-Net structure incorporating attention mechanisms, and the discriminator followed a patch-based design. Dr.Magic is intended to provide additional imaging information derived from CT to assist CT-based assessment of degenerative spinal disorders.

For inference, Dr.Magic operates in a fully automated manner without user interaction. Once a CT examination is provided, the system automatically processes the input and translates it into the corresponding $\hat{T}$-MRI image promptly. Radiologists remained responsible for the final diagnostic interpretation. Inference time was measured from CT input loading to completion of the $\hat{T}$-MRI output for each examination and is reported as median and IQR.

### 2.5. Reader Study and Evaluation Protocol

Three board-certified radiologists with more than 20 years of clinical experience independently participated in the reader study. Each reader evaluated all cases under three separate reading conditions: MRI-only, CT-only, and CT augmented with $\hat{T}$-MRI. MRI-only interpretation was performed first, during which readers had access only to MRI and no access to CT or $\hat{T}$-MRI information. CT-only interpretation was then performed on the same day, and readers were provided with CT images alone. CT interpretation augmented with $\hat{T}$-MRI was performed 2 weeks later, during which readers reviewed CT images together with the corresponding $\hat{T}$-MRI images. For each reading condition, the case presentation order was randomized separately. Readers did not have access to their interpretations from the other reading conditions and were blinded to the interpretations of the other readers.

MRI-only interpretation served as the reader-specific reference standard for each reader, and no consensus reading was performed. This per-reader reference standard approach was adopted because inter-reader variability has been reported in spine MRI interpretation for disc-related findings [20,21]. This design also enables paired comparisons within each reader across reading conditions while reflecting real-world interpretive variability.

Under each condition, readers (i) identified herniated nucleus pulposus (HNP), (ii) recorded the most severely affected intervertebral level (from L1/L2 through L5/S1), and (iii) assessed the presence or absence of degenerative disc changes at that level. Diagnostic performance was evaluated by comparing CT-only and CT augmented with $\hat{T}$-MRI interpretations to the reader-specific MRI-based reference standard. We defined accuracy using a composite correctness criterion: a case was counted as correct only when HNP status, the most severe level, and degenerative disc changes at that level were all concordant with the reader’s MRI-only interpretation. This composite correctness for case *i* under reading condition *c* was defined as

$${\mathrm{Correct}}_{i,c}=1\;\left[HNP_{i,c}=HNP_{i,\mathrm{MRI}}\;\wedge \;Level_{i,c}=Level_{i,\mathrm{MRI}}\;\wedge \;DDC_{i,c}=DDC_{i,\mathrm{MRI}}\right],$$

where 1[·] denotes the indicator function (1 if the condition holds, and 0 otherwise). The primary endpoint was diagnostic accuracy, defined by the composite correctness criterion described above. Component-wise analyses were also performed for HNP level assessment and degenerative disc change assessment. As an additional analysis, a majority-vote MRI reference was constructed from the three MRI-only interpretations for HNP diagnosis, HNP level, and degenerative disc change. CT-only and CT augmented with $\hat{T}$-MRI interpretations were then re-evaluated against this common reference. Two cases without majority agreement for HNP level were excluded from this analysis.

### 2.6. Statistical Analysis

Statistical analyses were performed using Python (version 3.10, Python Software Foundation) and statsmodels (version 0.14). The primary analysis compared paired diagnostic accuracy between CT-only and CT augmented with $\hat{T}$-MRI within each reader using McNemar’s test based on discordant pairs. Paired odds ratios (ORs) were calculated using discordant pairs from paired CT-only and CT augmented with $\hat{T}$-MRI results. Accuracy estimates were reported with 95% confidence intervals (CIs), which were computed using the Wilson method. Inter-reader agreement was assessed using Fleiss’ κ, with confidence intervals for κ and observed agreement ($P_{o}$) estimated by bootstrap resampling. A two-sided *p*-value < 0.05 was considered statistically significant.

## 3. Results

### 3.1. Diagnostic Accuracy Results

A total of 92 cases were evaluated by three radiologists under CT-only and CT augmented with $\hat{T}$-MRI conditions, using MRI-only interpretation as the reader-specific reference standard. Across all readers, CT interpretation augmented with $\hat{T}$-MRI yielded higher diagnostic accuracy than CT-only interpretation. For Reader 1, accuracy increased from 34.78% under CT-only to 72.83% under CT augmented with $\hat{T}$-MRI (p<0.01). For Reader 2, accuracy increased from 42.39% to 77.17% (p<0.01), and for Reader 3, from 40.22% to 69.57% (p<0.01). Overall, mean accuracy across readers increased from 39.13% ± 3.20% under CT-only to 73.19% ± 3.12% under CT augmented with $\hat{T}$-MRI. Paired ORs were also higher under CT augmented with $\hat{T}$-MRI than under CT-only across all readers. Detailed reader-level results are summarized in Table 2. In an additional analysis using a majority-vote MRI reference derived from the three MRI-only interpretations, two cases were excluded because no majority HNP level could be assigned from the MRI-only readings. The overall pattern of results remained unchanged, with significantly higher diagnostic accuracy under CT augmented with $\hat{T}$-MRI than under CT-only for all three readers and paired ORs showing the same direction of effect (Table S1).

### 3.2. Inter-Reader Agreement

Inter-reader agreement for the composite assessment of disc-related findings was quantified using Fleiss’ κ and observed agreement ($P_{o}$) for each reading condition. Fleiss’ κ values for MRI-only, CT-only, and CT augmented with $\hat{T}$-MRI were 0.5917 (95% CI, 0.47–0.70), 0.5617 (95% CI, 0.47–0.65), and 0.6621 (95% CI, 0.55–0.77), respectively. The corresponding $P_{o}$ values were 0.7572 (95% CI, 0.68–0.82), 0.6630 (95% CI, 0.59–0.74), and 0.8116 (95% CI, 0.75–0.87). Compared with CT-only, CT augmented with $\hat{T}$-MRI showed higher agreement (Δκ = +0.1005; $\Delta P_{o}$ = +0.1486). Agreement metrics across conditions are summarized in Table 3.

### 3.3. Component-Wise Diagnostic Performance

We additionally examined exact-level HNP assessment and degenerative disc change assessment separately to assess the component-wise pattern underlying the composite diagnostic accuracy. For exact-level HNP assessment, accuracy did not differ significantly between CT-only and CT augmented with $\hat{T}$-MRI for any reader (all p>0.05). However, inter-reader agreement for level assessment improved when $\hat{T}$-MRI was available, with Fleiss’ κ increasing from 0.5068 (95% CI, 0.39–0.62) under CT-only to 0.6692 (95% CI, 0.55–0.77) under CT augmented with $\hat{T}$-MRI, comparable to MRI-only interpretation (κ = 0.6686; 95% CI, 0.54–0.78).

In contrast, degenerative disc change assessment showed marked and consistent improvement under CT augmented with $\hat{T}$-MRI. Across readers, accuracy increased from 51.09%, 50.00%, and 54.35% under CT-only to 93.48%, 98.91%, and 91.30% under CT augmented with $\hat{T}$-MRI, respectively, and all improvements were statistically significant (all p<0.01).

This component-wise pattern was unchanged in an additional analysis using a majority-vote MRI reference, with no consistent improvement in exact-level HNP performance and persistently marked improvement in degenerative disc change assessment (Table S2).

### 3.4. Qualitative Comparison

**Figure 2:**
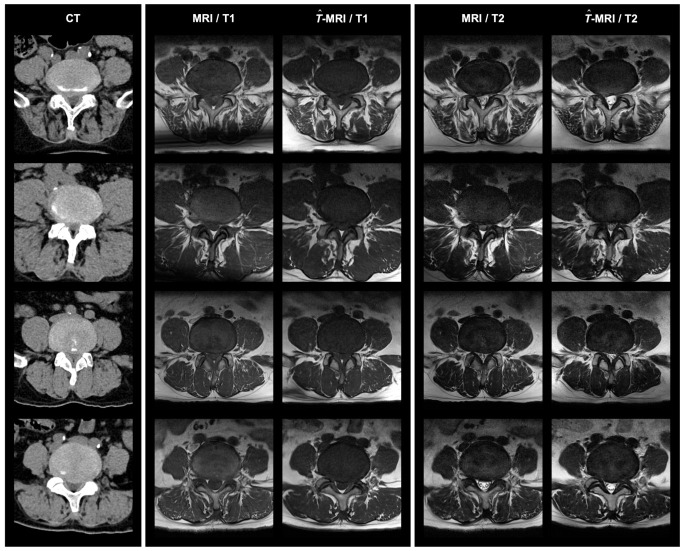
Representative axial slices comparing CT, MRI, and $\hat{T}$-MRI. Columns show CT, MRI/T1, $\hat{T}$-MRI/T1, MRI/T2, and $\hat{T}$-MRI/T2. Each row represents a different case at representative lumbar levels. $\hat{T}$-MRI is shown in the same spatial geometry as CT to support disc-related assessment.

**Figure 3:**
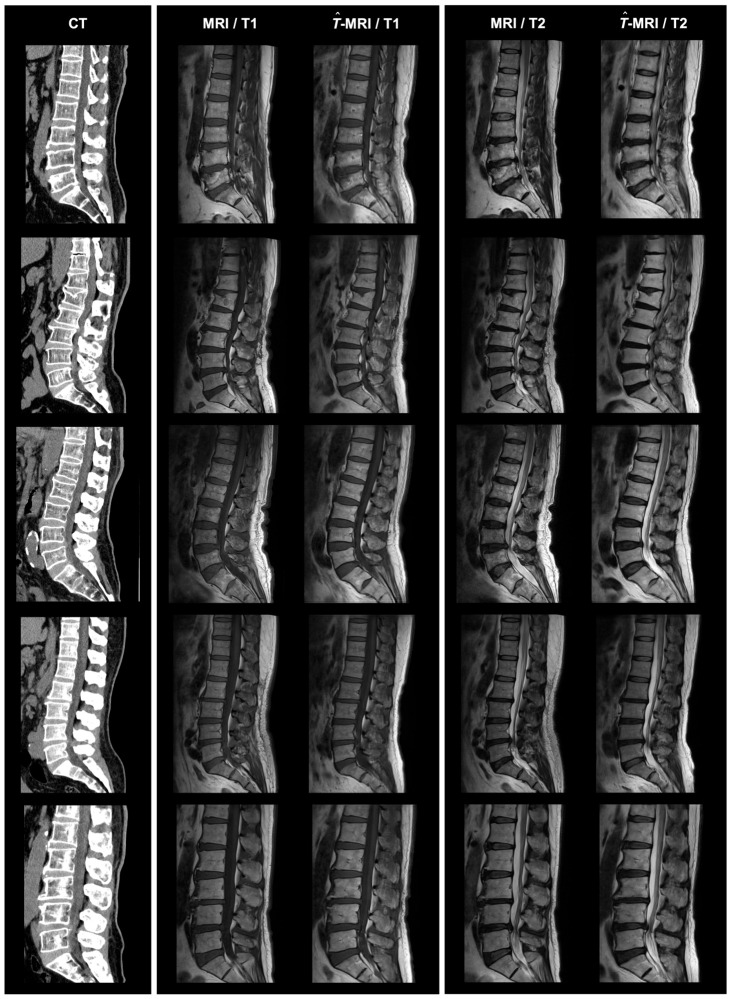
Representative sagittal slices comparing CT, MRI, and $\hat{T}$-MRI. Columns show CT, MRI/T1, $\hat{T}$-MRI/T1, MRI/T2, and $\hat{T}$-MRI/T2. Each row represents a different case. $\hat{T}$-MRI preserves CT-consistent anatomy while providing MRI-like soft-tissue contrast in qualitative visual assessment.

Figure 2 and Figure 3 show representative qualitative comparisons of CT, MRI, and $\hat{T}$-MRI. Because the present study was designed to evaluate reader interpretation of $\hat{T}$-MRI alongside CT rather than direct image-to-image comparison, CT and MRI were not spatially registered. Accordingly, the displayed CT and MRI slices were selected to represent approximately corresponding anatomical levels for qualitative visual comparison. In both axial and sagittal views, $\hat{T}$-MRI provided additional contrast for qualitative assessment of disc morphology and degenerative features.

## 4. Discussion

In this retrospective reader study, we evaluated CT interpretation augmented with $\hat{T}$-MRI from Dr.Magic (DRM-S-01) in comparison with CT-only interpretation for degenerative spinal disorder assessment. Following prior model development and technical evaluation, the present study was designed as a clinical reader study to assess how $\hat{T}$-MRI performs when used alongside CT in radiologist interpretation.

CT interpretation augmented with $\hat{T}$-MRI showed higher diagnostic performance than CT-only interpretation. Mean composite diagnostic accuracy across readers increased from 39.13% ± 3.20% under CT-only to 73.19% ± 3.12% under CT augmented with $\hat{T}$-MRI, and the improvement was statistically significant for all three readers (all p<0.01). Inter-reader agreement was higher when $\hat{T}$-MRI was available, suggesting greater consistency in interpretation. Component-wise analyses showed that the composite accuracy gain was driven primarily by improved degenerative disc change assessment, whereas exact-level HNP accuracy did not significantly change. Meanwhile, inter-reader agreement for HNP level assessment was higher when $\hat{T}$-MRI was available, suggesting greater consistency in level assignment even without a significant gain in exact-level accuracy. Thus, the observed composite accuracy gain appears to reflect primarily improved degenerative disc change assessment, together with greater consistency in HNP level assignment.

This pattern is noteworthy in spine imaging, where inter-reader variability is observed even on MRI. In our MRI-only readings, all three radiologists were in complete agreement in 60 of 92 cases (65.2%). In 29 cases (31.5%), two readers agreed and one disagreed, and in 3 cases (3.3%), all three readers disagreed, consistent with prior reports [20,21]. This supports the use of per-reader MRI-only assessments as individual reference standards in our study rather than reliance on a single consensus label. In addition, when we re-evaluated the data using a majority-vote MRI reference as an alternative single-reference definition, the overall pattern of improved performance under CT augmented with $\hat{T}$-MRI remained unchanged (Table S1). The same overall component-wise pattern was also preserved in the additional majority-vote MRI reference analysis (Table S2), supporting the robustness of the main findings and their interpretation.

From a workflow perspective, Dr.Magic provided $\hat{T}$-MRI in a median of 10.90 s (IQR, 10.39–11.79) per CT examination in our implementation, making additional image-derived information available almost immediately after CT acquisition. This timing may be advantageous in CT-based workflows when rapid triage or prioritization for subsequent MRI is needed, given that a conventional MRI examination typically requires approximately 30–60 min [22].

While the findings support the potential value of CT interpretation augmented with $\hat{T}$-MRI in degenerative spinal disorder assessment, several considerations should be noted. This study was conducted as a retrospective reader study at a single institution with a limited sample size. Because the cohort was drawn from patients undergoing imaging for evaluation of degenerative spinal disorders, it reflected an intended-use clinical cohort and was enriched for HNP-positive cases rather than representing a balanced negative or screening population. Accordingly, the present findings should be interpreted with caution when considering broader screening or general diagnostic settings. The potential influence of institutional imaging characteristics or local practice patterns cannot be excluded. External validation in larger, multi-center datasets is therefore needed to assess generalizability across more diverse imaging environments and patient populations. Cases with substantial metallic artifacts were excluded from both model development and the present study, because such artifacts could impair reliable interpretation and comparison between CT and MRI. Therefore, the current findings may not generalize to performance in artifact-affected examinations, and future work should investigate more robust approaches for handling metallic artifacts.

We used a per-reader MRI-only reference standard without consensus adjudication. This may be considered a limitation of the present study, because a single consensus-based reference was not established. However, this design was intended to reflect the real-world variability of spine MRI interpretation reported in prior studies [20,21], which was also observed in our MRI-only assessments. Future work should further evaluate this approach using consensus-adjudicated reference standards.

A 2-week washout period was used between CT-only and CT interpretation augmented with $\hat{T}$-MRI to reduce recall bias, consistent with prior imaging reader studies [23,24,25]. In addition, cases were presented in an independently randomized order for each reading condition, and interpretations from other conditions were not available during assessment. Nevertheless, residual memory effects cannot be fully excluded, and this design should be considered when interpreting the extent of the observed improvement.

The interval between CT and MRI should also be considered when interpreting the results. Although the inclusion criteria allowed intervals of up to 180 days, additional subgroup analysis showed the same overall direction of effect across the 0–30 day, 31–90 day, and 91–180 day interval groups, with higher diagnostic accuracy under CT augmented with $\hat{T}$-MRI than under CT-only in each subgroup (Table S3(A)). However, the longest-interval subgroup was small, and these findings should be interpreted cautiously.

The present study was designed to evaluate reader performance and qualitative visual utility when $\hat{T}$-MRI was interpreted alongside CT. GAN-based translation may introduce plausible but incorrect structures, and this risk should be considered when interpreting translated images. In the present study, this issue was assessed indirectly through reader-level diagnostic concordance with MRI-based interpretation rather than through direct image-level validation. The observed improvement in diagnostic concordance argues against a dominant effect of misleading or non-corresponding structures at the overall study level, although localized hallucination cannot be fully excluded. Accordingly, further studies in larger and more diverse cases are needed to establish confidence in the clinical safety of translated images. Further studies in larger and more diverse cases are needed to establish confidence in the safety and reliability of translated images. Future work should include dedicated image-level validation to assess anatomical fidelity and localized discrepancies in translated images, and may also investigate methods to provide case-level confidence information for $\hat{T}$-MRI output.

Additional transition-based analysis showed that CT-only incorrect to CT augmented with $\hat{T}$-MRI correct cases were substantially more frequent than CT-only correct to CT augmented with $\hat{T}$-MRI incorrect cases (110 vs. 16 reader-case pairs; Table S4). Most improvements were attributable to correction of degenerative disc change assessment, whereas most worsening cases involved HNP level mislocalization. Representative improved and worsened cases are provided in Figures S1 and S2 to illustrate typical patterns of HNP level assignment change, degenerative disc change correction, and potential localized misleading interpretation.

Subgroup analysis according to the degree of agreement among MRI-only interpretations showed that the improvement with CT augmented with $\hat{T}$-MRI was most pronounced in cases with unanimous MRI-only agreement. Cases without clear agreement on MRI-only interpretation remained difficult, suggesting that intrinsically ambiguous cases may require further study even when $\hat{T}$-MRI is available (Table S3(B)).

From a clinical workflow perspective, these findings support the potential utility of Dr.Magic (DRM-S-01) for degenerative spinal disorder assessment when timely MRI is not feasible. By providing $\hat{T}$-MRI alongside CT, radiologists may perform more reliable disc-related assessment and reach more consistent interpretations, while remaining responsible for final diagnosis and management decisions. Dr.Magic may be particularly relevant in settings where MRI access is delayed or limited, or when rapid decision-making is required within CT-based workflows.

## 5. Conclusions

In this retrospective reader study, CT interpretation augmented with $\hat{T}$-MRI provided by Dr.Magic (DRM-S-01) improved composite diagnostic accuracy for degenerative spinal disorder assessment compared with CT-only interpretation. Mean composite diagnostic accuracy across three radiologists increased from 39.13% ± 3.20% under CT-only to 73.19% ± 3.12% under CT augmented with $\hat{T}$-MRI, and the improvement was statistically significant for each reader (all p<0.01). Reading consistency was also higher when $\hat{T}$-MRI was available (Fleiss’ κ, 0.5617 to 0.6621). In our implementation, $\hat{T}$-MRI became available in a median of 10.90 s per CT examination (IQR, 10.39–11.79), supporting its potential utility in CT-based workflows when rapid triage or prioritization for subsequent MRI is needed.

## Figures and Tables

**Table 1:** Baseline characteristics of the study population, including demographic information and the interval between CT and MRI examinations.

Characteristic	Statistic	Value
Age (years)	Mean ± SD	68.9 ± 9.6
Sex	*n* (%)	Male 38 (41.3); Female 54 (58.7)
CT–MRI interval (days)	Median (IQR)	17 (1–37.5)

**Table 2:** Diagnostic accuracy of CT-only and CT augmented with $\hat{T}$-MRI using the reader-specific MRI reference standard.

Reader	CT-Only Accuracy, % (95% CI)	CT Augmented with $\hat{T}$-MRI Accuracy, % (95% CI)	Δ (pp)	Paired OR (95% CI)	*p*-Value *
1	34.78 (25.84–44.95)	72.83 (62.96–80.86)	38.04	7.364 (3.022–17.943)	<0.01
2	42.39 (32.80–52.59)	77.17 (67.61–84.56)	34.78	6.818 (2.786–16.685)	<0.01
3	40.22 (30.78–50.43)	69.57 (59.54–78.03)	29.35	5.154 (2.225–11.939)	<0.01
Mean ± SD	39.13 ± 3.20	73.19 ± 3.12	34.06 ± 3.59	-	-

* *p*-values were obtained using McNemar’s test for paired binary outcomes.

**Table 3:** Inter-reader agreement for the composite assessment of disc-related findings across reading conditions. Composite categories were defined by the combination of HNP presence, most severe intervertebral level, and degenerative disc change at that level.

Reading Condition	Fleiss’ κ (95% CI)	Observed Agreement (95% CI)
MRI-only	0.5917 (0.47–0.70)	0.7572 (0.68–0.82)
CT-only	0.5617 (0.47–0.65)	0.6630 (0.59–0.74)
CT augmented with $\hat{T}$-MRI	0.6621 (0.55–0.77)	0.8116 (0.75–0.87)

## Data Availability

The data supporting the findings of this study are available upon reasonable request from the author (Hyung-Youl Park). Access is restricted due to ethical considerations and privacy concerns.

## Supplementary Materials

The following supporting information can be downloaded at: https://www.mdpi.com/article/10.3390/diagnostics16111589/s1, Table S1. Diagnostic accuracy using a majority-vote MRI reference derived from the three MRI-only interpretations. Accuracy under CT-only and CT augmented with $\hat{T}$-MRI was recalculated against a common MRI-based reference. Two cases without majority agreement for MRI-only HNP level were excluded. Table S2. Component-wise diagnostic accuracy using two MRI-based reference definitions. (A) shows results using a reader-specific MRI reference, and (B) shows results using a majority-vote MRI reference. Table S3. Robustness subgroup analyses of composite diagnostic accuracy. (A) shows accuracy according to the CT-MRI interval subgroup. (B) shows accuracy according to the degree of agreement among the three MRI-only interpretations, categorized as unanimous, 2-of-3 agreement, and 3-way disagreement. Accuracy values are reported at the reader-case pair level. Table S4. Transition-based failure analysis of composite diagnostic correctness. (A) summarizes readercase pair classification changes between CT-only and CT augmented with $\hat{T}$-MRI relative to the MRIbased reference. (B) shows the error-type breakdown of improved and worsened cases according to whether changes were attributable to HNP level assessment, degenerative disc change assessment, or both. Figure S1. Representative improved cases under CT augmented with $\hat{T}$-MRI. Figure S2. Representative worsened cases under CT augmented with $\hat{T}$-MRI.

## Abbreviations

The following abbreviations are used in this manuscript:

CT	Computed tomography
MRI	Magnetic resonance imaging
AI	Artificial intelligence
HNP	Herniated nucleus pulposus
