# Supplementary material for: Clinical Utility of AI-Enabled CT-to-MRI Translation for Degenerative Spinal Disorders: A Retrospective Reader Study
Source: Diagnostics (Basel). 2026 May 22;16(11):1589. doi: 10.3390/diagnostics16111589 (PMC13257241; doi:10.3390/diagnostics16111589)
Supplement: Supplementary file 1 [file diagnostics-16-01589-s001.zip › diagnostics-4251844-supplementary.pdf]

Supplementary Materials

1 Supplementary Tables

Table S1. Diagnostic accuracy using a majority-vote MRI reference derived from the three MRI-only interpretations. Accuracy under CT-only and CT augmented with $\hat{T}$-MRI was recalculated against a common MRI-based reference. Two cases without majority agreement for MRI-only HNP level were excluded.

**Reader**	**CT-only accuracy, % (95% CI)**	**CT augmented with $\hat{T}$-MRI accuracy, % (95% CI)**	**Δ (pp)**	**Paired OR (95% CI)**	**P-value**
1	35.56 (26.44–45.85)	75.56 (65.75–83.27)	40.00	9.000 (3.398–23.835)	< 0.01
2	40.00 (30.49–50.33)	75.56 (65.75–83.27)	35.56	6.818 (2.786–16.685)	< 0.01
3	38.89 (29.47–49.22)	77.78 (68.15–85.13)	38.89	11.000 (3.683–32.856)	< 0.01
Mean ±SD	38.15 ±1.89	76.30 ±1.05	38.15 ±1.89	-	-

Two cases were excluded from the majority-vote analysis because no majority HNP level could be assigned from the three MRI-only interpretations.

Table S2. Component-wise diagnostic accuracy using two MRI-based reference definitions. (A) shows results using a reader-specific MRI reference, and (B) shows results using a majority-vote MRI reference.

(A) Reader-specific MRI reference

Component	Reader	CT-only accuracy, % (95% CI)	CT augmented with $\hat{T}$-MRI accuracy, % (95% CI)	P-value
**HNP level assessment**	1	65.22 (55.05–74.16)	75.00 (65.27–82.72)	0.09
	2	85.87 (77.31–91.55)	77.17 (67.61–84.56)	0.08
	3	75.00 (65.27–82.72)	75.00 (65.27–82.72)	1.00
**Degenerative disc change assessment**	1	51.09 (41.04–61.05)	93.48 (86.49–96.98)	< 0.01
	2	50.00 (39.99–60.01)	98.91 (94.10–99.81)	< 0.01
	3	54.35 (44.20–64.15)	91.30 (83.77–95.53)	< 0.01

(B) Majority-vote MRI reference

Component	Reader	CT-only accuracy, % (95% CI)	CT augmented with $\hat{T}$-MRI accuracy, % (95% CI)	P-value
**HNP level assessment**	1	64.44 (54.15–73.56)	76.67 (66.95–84.20)	0.03
	2	81.11 (71.82–87.86)	77.78 (68.15–85.13)	0.61
	3	75.56 (65.75–83.27)	80.00 (70.59–86.96)	0.34
**Degenerative disc change assessment**	1	52.22 (42.02–62.24)	95.56 (89.12–98.26)	< 0.01
	2	50.00 (39.88–60.12)	96.67 (90.65–98.86)	< 0.01
	3	51.11 (40.95–61.18)	95.56 (89.12–98.26)	< 0.01

Table S3. Robustness subgroup analyses of composite diagnostic accuracy. (A) shows accuracy according to the CT–MRI interval subgroup. (B) shows accuracy according to the degree of agreement among the three MRI-only interpretations, categorized as unanimous, 2-of-3 agreement, and 3-way disagreement. Accuracy values are reported at the reader-case pair level.

(A) CT–MRI interval subgroup analysis

Group	Reader-case pairs, n	CT-only accuracy, % (95% CI)	CT augmented with $\hat{T}$-MRI accuracy, % (95% CI)
0–30 days	192	39.06 (32.44–46.11)	70.83 (64.05–76.80)
31–90 days	60	46.67 (34.63–59.11)	80.00 (68.22–88.17)
91–180 days	24	20.83 (9.24–40.47)	75.00 (55.10–88.00)

(B) MRI-only agreement subgroup analysis

Group	Reader-case pairs, n	CT-only accuracy, % (95% CI)	CT augmented with $\hat{T}$-MRI accuracy, % (95% CI)
Unanimous	180	41.11 (34.18–48.41)	86.67 (80.93–90.87)
2-of-3 agreement	87	35.63 (26.37–46.11)	50.57 (40.27–60.83)
3-way disagreement	9	33.33 (12.06–64.58)	22.22 (6.32–54.74)

MRI-only agreement subgroups were defined according to agreement among the three readers on the MRI-only composite interpretation, which comprised HNP status, the most severely affected level, and degenerative disc change at that level. Reader-case pairs represent the unit of analysis and were defined as one interpretation by one reader for one case; therefore, each case contributed three reader-case pairs. The MRI-only agreement subgroups comprised 60 cases with unanimous agreement, 29 cases with 2-of-3 agreement, and 3 cases with 3-way disagreement. The CT–MRI interval subgroups comprised 64 cases for 0–30 days, 20 cases for 31–90 days, and 8 cases for 91–180 days.

Table S4. Transition-based failure analysis of composite diagnostic correctness. (A) summarizes reader-case pair classification changes between CT-only and CT augmented with $\hat{T}$-MRI relative to the MRI-based reference. (B) shows the error-type breakdown of improved and worsened cases according to whether changes were attributable to HNP level assessment, degenerative disc change assessment, or both.

(A) Transition summary

**Transition category**	**n**	**%**
Improved	110	39.86
Worsened	16	5.80
Persistently incorrect	58	21.01
Persistently correct	92	33.33

(B) Error-type breakdown

**Transition category**	**Error type**	**n**	**%**
**Improved**	HNP level only	11	10.00
	DDC only	87	79.09
	Both	12	10.91
**Worsened**	HNP level only	13	81.25
	DDC only	3	18.75
	Both	0	0.00

Improved indicates CT-only incorrect and CT augmented with $\hat{T}$-MRI correct; *Worsened* indicates CT-only correct and CT augmented with $\hat{T}$-MRI incorrect; *Persistently incorrect* indicates both CT-only and CT augmented with $\hat{T}$-MRI incorrect; *Persistently correct* indicates both CT-only and CT augmented with $\hat{T}$-MRI correct.

2 Supplementary Figures

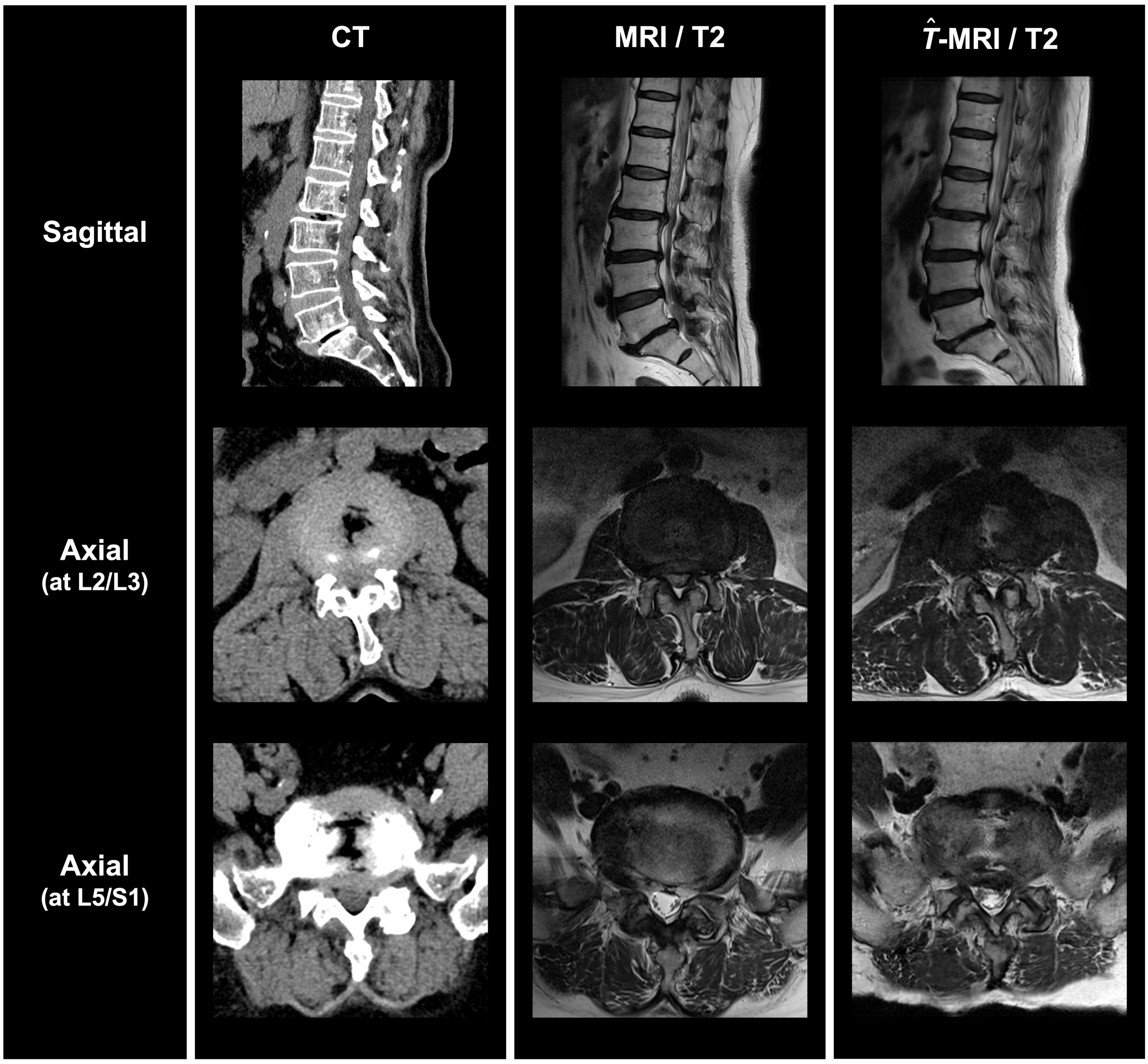

(a) Improved HNP level assignment. Under MRI-only, the most severely affected level was interpreted as L2/L3; under CT-only, it was interpreted as L5/S1; and under CT augmented with $\hat{T}$-MRI, it was interpreted as L2/L3. Sagittal images and corresponding axial images at L2/L3 and L5/S1 are shown.

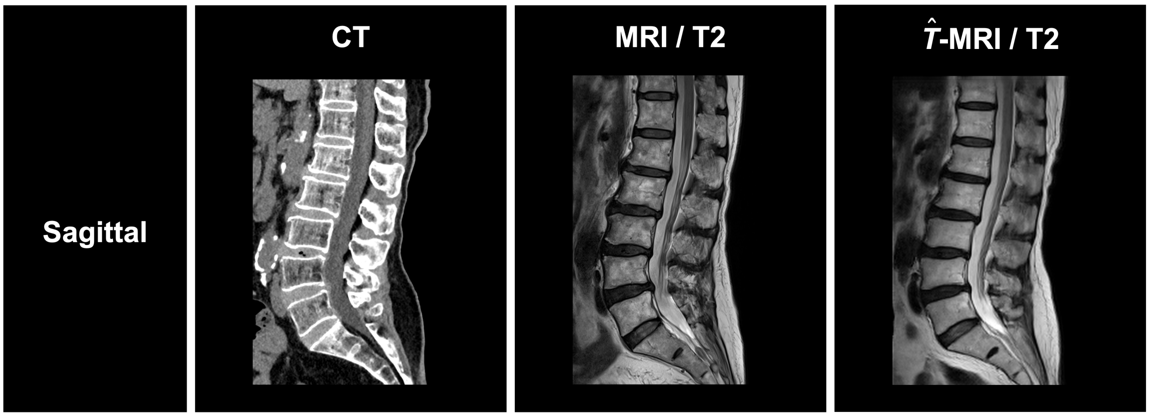

(b) Improved degenerative disc change assessment. Under CT-only, degenerative disc change assessment was discordant with the MRI-only interpretation, whereas under CT augmented with $\hat{T}$-MRI, it became concordant. Sagittal CT, T2-weighted MRI, and T2-like $\hat{T}$-MRI images are shown.

Figure S1. Representative improved cases under CT augmented with $\hat{T}$-MRI.

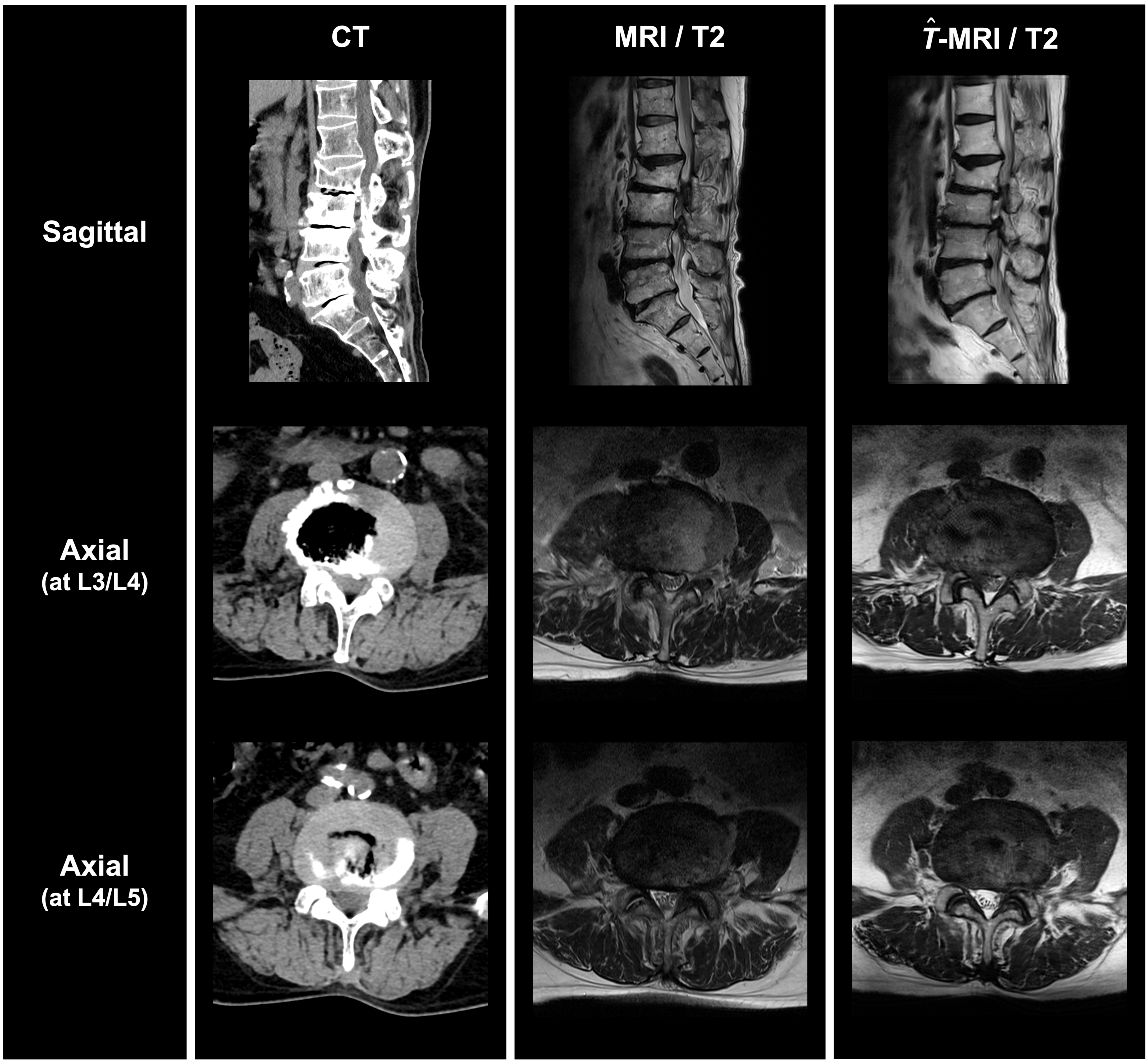

(a) Worsened HNP level assignment. Under MRI-only and CT-only, the most severely affected level was interpreted as L4/L5, whereas under CT augmented with $\hat{T}$-MRI, it was interpreted as L3/L4. Sagittal images and corresponding axial images at L3/L4 and L4/L5 are shown.

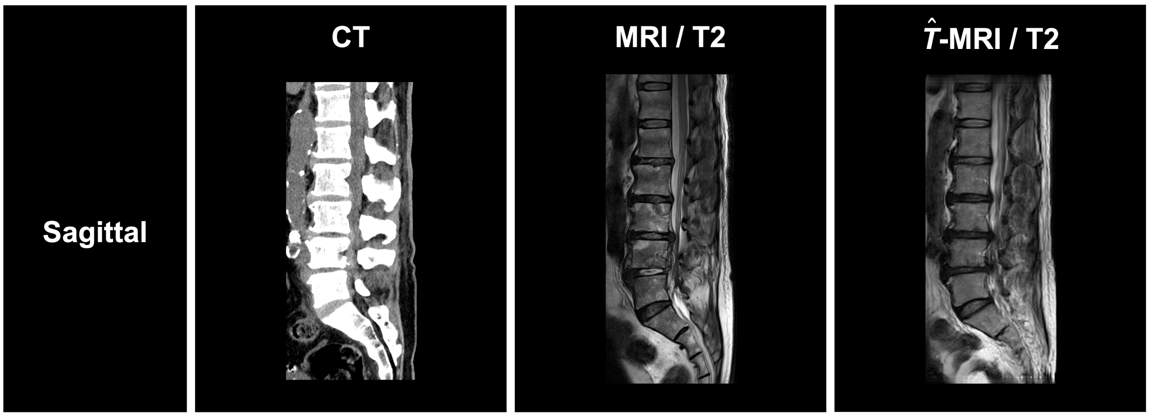

(b) Worsened degenerative disc change assessment. Under CT-only, degenerative disc change assessment was concordant with the MRI-only interpretation, whereas under CT augmented with $\hat{T}$-MRI, it became discordant. Sagittal CT, T2-weighted MRI, and T2-like $\hat{T}$-MRI images are shown.

Figure S2. Representative worsened cases under CT augmented with $\hat{T}$-MRI.
